# The role of anti-aquaporin 4 antibody in the conversion of acute brainstem syndrome to neuromyelitis optica

**DOI:** 10.1186/s12883-016-0721-1

**Published:** 2016-10-21

**Authors:** Chen Cheng, Ying Jiang, Xiaodong Lu, Fu Gu, Zhuang Kang, Yongqiang Dai, Zhengqi Lu, Xueqiang Hu

**Affiliations:** 1 Department of Neurology, The Affiliated Hospital, Hangzhou Normal University, 126 Wenzhou Road, Hangzhou, Zhejiang 310015 People’s Republic of China; 2 Department of Neurology, The Third Affiliated Hospital, Sun Yat-sen University, 600 Tianhe Road, Guangzhou, Guangdong 510630 People’s Republic of China; 3 Department of Chemical and Environmental Engineering, University of Nottingham, 199 Taikang East Road, Ningbo, Zhejiang 315100 People’s Republic of China; 4 Department of Radiology, the Third Affiliated Hospital, Sun Yat-sen University, 600 Tianhe Road, Guangzhou, Guangdong 510630 People’s Republic of China

**Keywords:** Acute brainstem syndrome, Anti-aquaporin 4 antibody, Neuromyelitis optica, Magnetic resonance imaging

## Abstract

**Background:**

Acute brainstem syndrome (ABS) may herald multiple sclerosis (MS), neuromyelitis optica (NMO), or occur as an isolated syndrome. The aquaporin 4 (AQP4)-specific serum autoantibody, NMO-IgG, is a biomarker for NMO. However, the role of anti-AQP4 antibody in the conversion of ABS to NMO is unclear.

**Methods:**

Thirty-one patients with first-event ABS were divided into two groups according to the presence of anti-AQP4 antibodies, their clinical features and outcomes were retrospectively analyzed.

**Results:**

Fourteen of 31 patients (45.16 %) were seropositive for NMO-IgG. The 71.43 % of anti-AQP4 (+) ABS patients converted to NMO, while only 11.76 % of anti-AQP4 (-) ABS patients progressed to NMO. Anti-AQP4 (+) ABS patients demonstrated a higher IgG index (0.68 ± 0.43 vs 0.42 ± 0.13, *p* < 0.01) and Kurtzke Expanded Disability Status Scale (4.64 ± 0.93 vs 2.56 ± 0.81, *p* < 0.01) than anti-AQP4 (-) ABS patients. Area postrema clinical brainstem symptoms occurred more frequently in anti-AQP4 (+) ABS patients than those in anti-AQP4 (-) ABS patients (71.43 % vs 17.65 %, *p* = 0.004). In examination of magnetic resonance imaging (MRI), the 78.57 % of anti-AQP4 (+) ABS patients had medulla-predominant involvements in the sagittal view and dorsal-predominant involvements in the axial view.

**Conclusions:**

ABS represents an inaugural or limited form of NMO in a high proportion of anti-AQP4 (+) patients.

## Background

Acute brainstem syndrome (ABS) is an acute inflammatory demyelinating syndrome of the CNS that may occur in isolation or herald multiple sclerosis (MS), neuromyelitis optica (NMO), or recurrences of brainstem syndrome without other CNS manifestation (idiopathic recurrent brainstem encephalitis [RBE]) [1]. Typical NMO is defined by attacks of myelitis and optic neuritis (ON) [2]. The aquaporin 4 (AQP4)-specific serum autoantibody, NMO-IgG, is recognized as a specific biomarker for NMO [3]. In NMO patients, ABS is untypical and not easily detected by physicians [4–7] and brainstem symptoms commonly presented as nausea, vomit, intractable hiccup [8–15], so ABS is easily neglected in NMO patients. In some cases of NMO, ABS acts as the first manifestation, which should be considered as a part of disease in addition to ON and myelitis [4, 12, 13, 16].

ABS is recognized more frequently in patients with NMO spectrum disorder (NMOSD), most brainstem symptoms exist even before the diagnosis of NMOSD [10–12, 17]. In diagnostic criteria of NMOSD in 2015, ABS is considered as one of the core clinical characteristics in the diagnosis of NMOSD [1]. In this study, the predictive value of anti-AQP4 antibody for relapse or later development was tested after the first sign of ABS.

## Methods

### Study population

Thirty-one patients with first-event ABS admitted to the Third Affiliated Hospital of Sun Yat-sen University in Guangzhou, China from January 2009 to September 2011 were retrospectively analyzed. All the patients involved in this study fulfilled the following inclusive criteria: 1) test for NMO-IgG; 2) single clinical episode of ABS associated with relevant brainstem MRI lesions 3) no other neurologic signs or symptoms which suggested the diagnosis of MS or NMO before NMO-IgG testing. Exclusion criteria included previous or concomitant systemic autoimmune diseases, metabolic etiology, vascular disorders and infections. All the patients were negative for HIV antibody.

The subjects provided written informed consent. This study was conducted according to the principles expressed in the Declaration of Helsinki and approved by the institution’s ethics committee. Lumbar puncture was also performed with informed consent.

### Data collection

Clinical features and outcomes including gender, age at onset, duration, relapse times, annualized recurrence rate, clinical manifestations, and magnetic resonance imaging (MRI) findings were recorded in details. Analysis of cerebrospinal fluid (CSF), serum anti-AQP4 test and examinations of magnetic resonance imaging (MRI) were performed within 2 weeks after attack of brainstem symptoms and before treatment. The diagnosis of NMO, NMOSD and MS was based on Wingerchuk’s criteria in 2006 [18], International consensus diagnostic criteria for NMOSD in 2015 [1] and McDonald’s criteria in 2010 [19] respectively. These symptoms, such as area postrema clinical syndrome (including intractable hiccups, nausea and vomiting), diplopia, and bulbar dysfunctions, were regarded as the manifestation of brainstem symptoms. A relapse of ABS was defined as definite brainstem symptoms of neurological dysfunction that lasted more than 24 h, together with relevant brainstem lesions after ruling out infective agents. Symptoms occurring within 1 month after the initial symptoms of relapse were considered to be part of the same episode. The neurological disability of the patients was assessed using the Kurtzke Expanded Disability Status Scale (EDSS) [20]. A corticosteroid (1000 mg methyl prednisone, administered intravenously for five consecutive days) was prescribed in the acute stage and some patients received azathioprine in the remission stage when developed as NMO.

### AQP4 and oligoclonal bands testing

AQP4 antibodies were determined using a cell-based assay on an AQP4-transfected cell line from a commercial BIOCHIP kit (EUROIMMUN AG, Lübeck, Germany) according to the manufacturer’s instructions. The CSF oligoclonal bands (OCB) detection method used in our laboratory was an isoelectric focusing technique combined with the avidin-biotinperoxidase complex method.

### MRI scanning

Brain and spinal cord MRI scans were carried out for all patients using a GE 1.5 T MR scanner (General Electric, Milwaukee, WI, USA). The slice thickness of the axial scans was between 3 and 5 mm. Conventional MRI protocols were used: T1 with and without gadolinium enhancement (400/15.5 ms, TR/TE) and T2 (2500–3500/100 ms, TR/TE) in spinal cord MRI; and T1 with and without gadolinium enhancement (2128–2300/11.6–12.4 ms, TR/TE), T2 (4600–4640/97.8–102 ms, TR/TE), and fluid-attenuated inversion recovery (FLAIR) (8800/120 ms, TR/TE) in brain MRI. A cross-sectional evaluation was also performed on all MRI scans of the brain, and brainstem lesions were classified as either having a ventral pattern, a dorsal pattern. The spinal cord was segmented into cervical and thoracolumbar regions. LETM is a spinal cord lesion that extends over 3 or more vertebral segments. All MRI scans were carried out prior to use of corticosteroid, immunomodulatory or immunosuppressive treatment. An experienced neuroradiologist and a neurologist, both of whom were blinded to the diagnostic categorization and the patients’ clinical features, each analyzed all of the MRI scans. The final assessments were made by consensus.

### Statistical analysis

All statistical analyses were performed using Statistical Program for Social Sciences (SPSS) statistical software (version 16.0; Chicago, IL, USA). For each set of values, data were expressed as the means ± standard deviation (SD). Categorical variables were compared using Fisher’s exact test. Non-categorical variables were compared using the Mann-Whitney *U* test. Survival was estimated according to the Kaplan-Meier method. The primary study endpoint was the time to NMO conversion, as indicated by the Wingerchuk’s criteria [18]. The log-rank test was used to compare the survival analysis between anti-AQP4 (+) and anti-AQP4 (-) ABS patients. All *p*-values were two-tailed, with values of < 0.05 considered significant. This study was an exploratory study so that no adjustment for multiple comparisons was made.

## Results

**Fig. 1:**
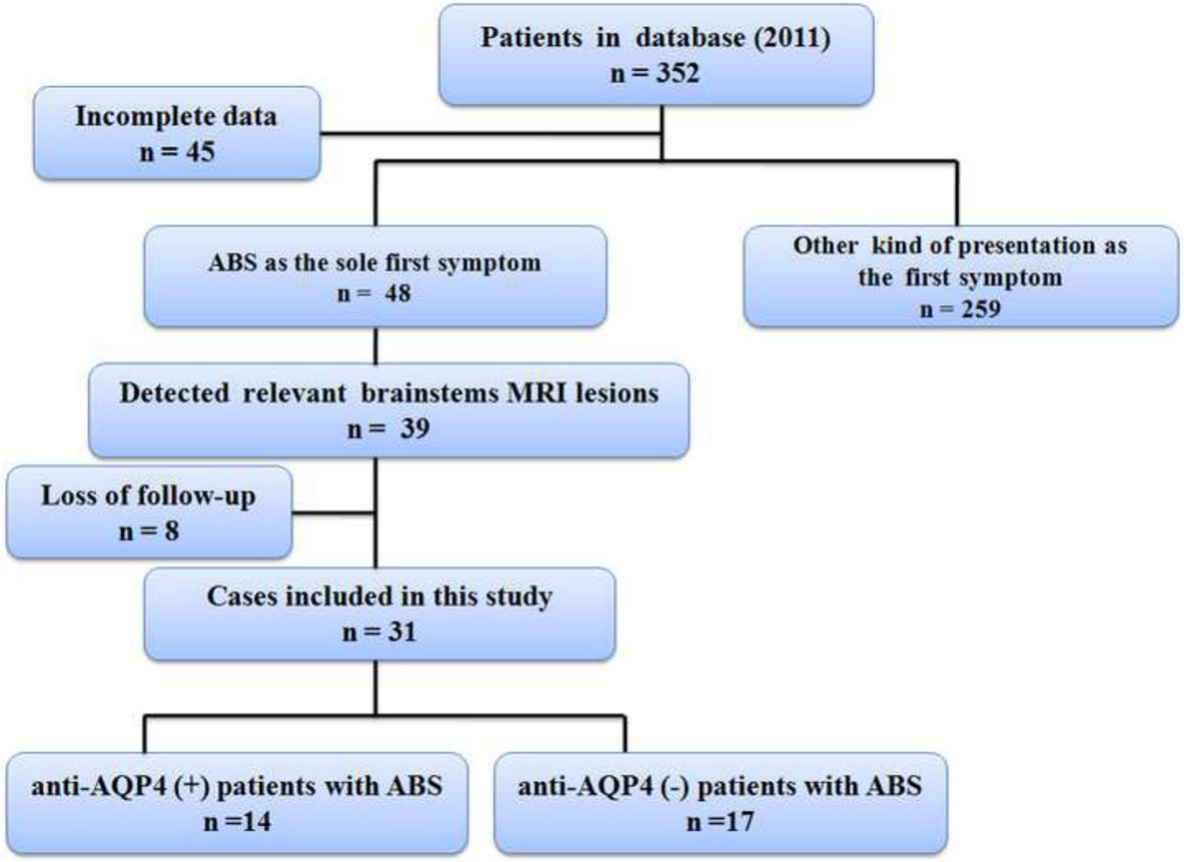
Study flowchart

The data of 352 patients in our database were reviewed between 2009 and 2011. A total of 31 patients who were enrolled in this study satisfied the diagnostic criteria: 14 anti-AQP4 (+) patients with ABS and 17 anti-AQP4 (-) patients with ABS. The details of the enrollment process can be seen in the flowchart (Fig. 1).

### Clinical characteristics

In 31 ABS patients (female-male ratio is 5.2:1) 14 (45.16 %) were positive for anti-AQP4 antibodies (Table 1). The mean age of onset for anti-AQP4 (+) ABS patients (31.21 ± 8.81 years) was similar to that of anti-AQP4 (-) patients (30.82 ± 8.55 years). According to Wingerchuk criteria in 2006, 71.43 % anti-AQP4 (+) patients (10/14) converted to NMO while only 11.76 % anti-AQP4 (-) ABS patients (2/17) converted to NMO within the follow-up after first-event brainstem symptoms (*p* < 0.01). The 100 % anti-AQP4 (+) patients (14/14) converted to NMOSD while only 17.65 % anti-AQP4 (-) ABS patients (3/17) converted to NMOSD (*p* < 0.001) according to International consensus diagnostic criteria for NMOSD in 2015. The cumulative NMO conversion probability was significantly higher in anti-AQP4 (+) patients than in anti-AQP4 (-) patients (Table 1, OR 18.75; 95 % CI 2.871 to 122.452; *p* = 0.001).

**Table 1:** Comparison of clinical features between anti-AQP4 (+) and anti-AQP4 (-) patients with ABS

Clinical features	Anti-AQP4 (+) *n* = 14	Anti-AQP4 (-) *n* = 17	Total *n* = 31	*P* value
Female/male ratio	1:13	4:13	5:26	0.344
Age of onset (±SD) (years)	31.21 ± 8.81	30.82 ± 8.55	31.00 ± 8.52	0.902
Duration of follow-up (months)	46.35 ± 13.80	40.00 ± 11.30	44.51 ± 14.86	0.169
Number of episodes	3.71 ± 0.73	2.41 ± 1.54	3.00 ± 1.39	0.005
Annual relapse rate	1.05 ± 0.40	0.72 ± 0.40	0.87 ± 0.43	0.031
NMOSD	14/14 (100 %)	3/17 (17.65 %)	17/31 (54.84 %)	<0.001
NMO	10/14 (71.43 %)	2/17 (11.76 %)	12/31 (38.71 %)	0.001
MS	0/14 (0.00 %)	7/17 (41.17 %)	7/31 (22.58 %)	0.007
Monophasic	0/14 (0.00 %)	7/17 (41.17 %)	7/31 (22.58 %)	0.007
CSF examinations				
Total protein (mg/dl, ±SD)	0.30 ± 0.10	0.25 ± 0.12	0.27 ± 0.11	0.204
Cell counts (number/μl, ±SD)	11.29 ± 4.27	8.47 ± 2.07	9.74 ± 2.21	0.07
Pleocytosis >50WBC/μl (%)	2/14	0/17	2/31	0.196
IgG index	0.68 ± 0.07	0.42 ± 0.13	0.54 ± 0.17	<0.01
Oligoclonal bands	1/14 (7.14 %)	1/17 (5.88 %)	2/31 (6.45 %)	0.708
EDSS a	3 (2–4)	3 (2–3)	3 (2–4)	0.141
EDSS b	5 (3–7)	2.5 (1.5–4.5)	3.5 (1.5–7)	<0.001

*ABS* acute brainstem syndrome, *NMO* neuromyelitis optica, *NMOSD* neuromyelitis optica spectrum disorder, *MS* multiple Sclerosis, *ON* optic neuritis, *CSF* cerebrospinal fluid, *EDSS* Kurtzke Expanded Disability Status Scale, a, EDSS scores at the first attack; b, EDSS scores of the last visit of follow-up

### Brainstem symptoms

The brainstem symptoms of 31 ABS patients were listed in Table 2. Five anti-AQP4 (+) patients (35.71 %) experienced recurrent brainstem symptoms before the attacks of ON or myelitis, while only one anti-AQP4 (-) patients (5.88 %, *p* = 0.05) had more than one brainstem attacks. Area postrema clinical brainstem symptoms, such as hiccup (29.41 % vs 5.88 %, *p* = 0.05), nausea and vomiting (64.28 % vs 17.65 %, *p* = 0.011), occurred more frequently in anti-AQP4 (+) patients than those in anti-AQP4 (-) patients.

**Table 2:** Comparison of brainstem symptoms between anti-AQP4 (+) and anti-AQP4 (-) patients with ABS

Onset symptoms	Anti-AQP4 (+)	Anti-AQP4 (-)	Total	*P* value
	*n* = 14	*n* = 17	*n* = 31	
Diplopia	6/14 (42.86 %)	8/17 (47.06 %)	14/31 (45.16 %)	0.551
Area postrema clinical syndrome	10/14 (71.43 %)	3/17 (17.65 %)	13/31 (41.94 %)	0.004
Intractable hiccup	5/14 (29.41 %)	1/17 (5.88 %)	6/31 (19.35 %)	0.05
Nausea and vomiting	9/14 (64.28 %)	3/17 (17.65 %)	12/31 (38.71 %)	0.011
Bulbar dysfunction	5/14 (35.71 %)	6/17 (35.29 %)	11/31 (35.48 %)	0.636
Dysarthria	1/14 (7.14 %)	3/17 (17.65 %)	4/31 (12.90 %)	0.378
Dysphagia	2/14 (14.29 %)	4/17 (23.53 %)	6/31 (19.35 %)	0.429
Alalia	2/14 (14.29 %)	0/17 (0 %)	2/31 (6.45 %)	0.196
Vertigo	4/14 (28.57 %)	7/17 (41.18 %)	11/31 (35.48 %)	0.364
Facial paralysis	2/14 (14.29 %)	5/17 (29.41 %)	7/31 (22.58 %)	0.287
Ataxia	3/14 (21.43 %)	7/17 (41.18 %)	7/31 (22.58 %)	0.218
Quadriplegia	2/14 (14.29 %)	1/17 (5.88 %)	3/31 (9.68 %)	0.425

*ABS* acute brainstem syndrome

### MRI examinations

**Fig. 2:**
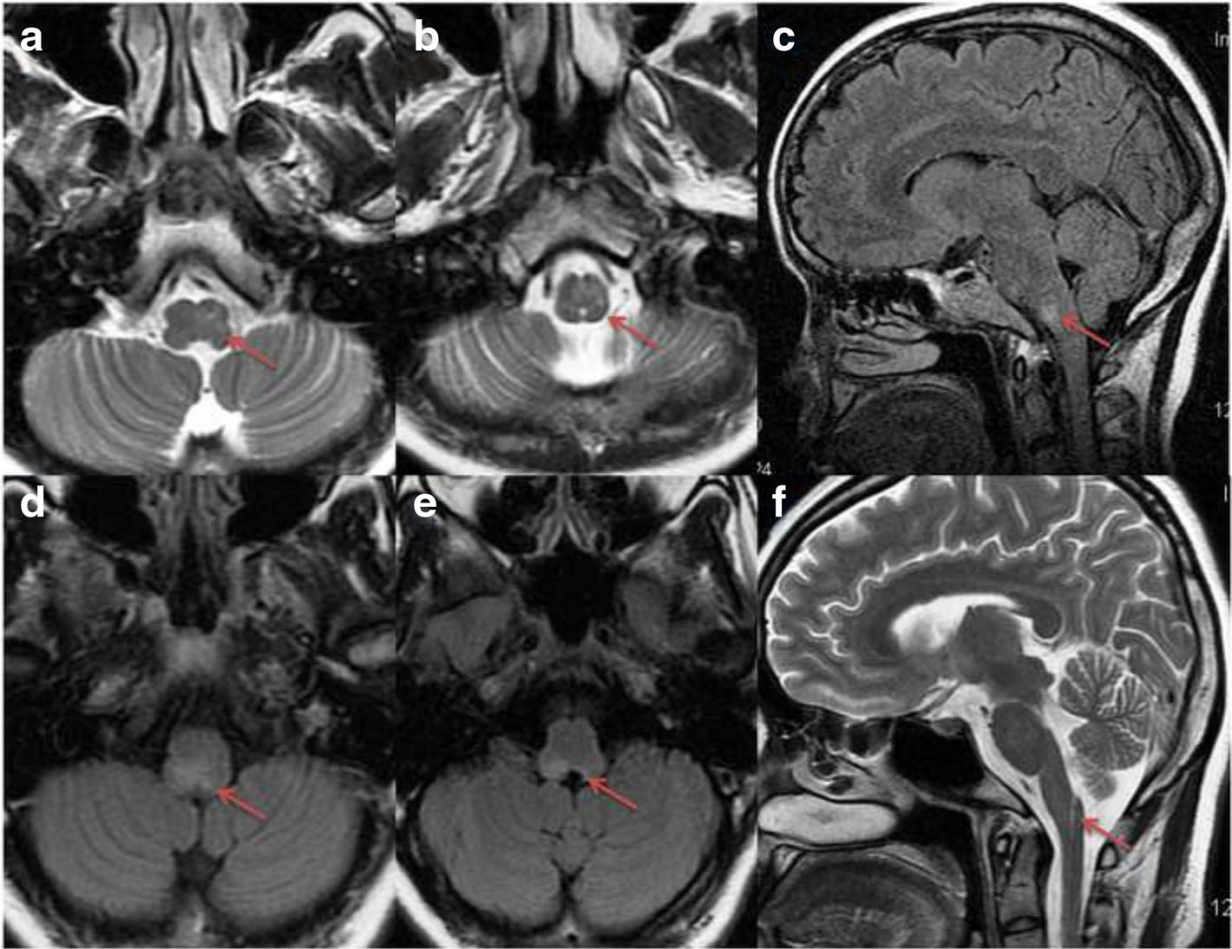
Axial T2-weighted (**a** and **b**; *arrow*) and sagittal T2 FLAIR (**c**; *arrow*) MRI show lesions occur in the medulla oblongata. Axial T2 FLAIR (**d** and **e**; *arrow*) MRI show pericanal lesions occur in the medulla oblongata. Linear faint Sagittal-T2-weighted high intensity signals are shown in the medulla oblongata (**f**; *arrow*)

**Fig. 3:**
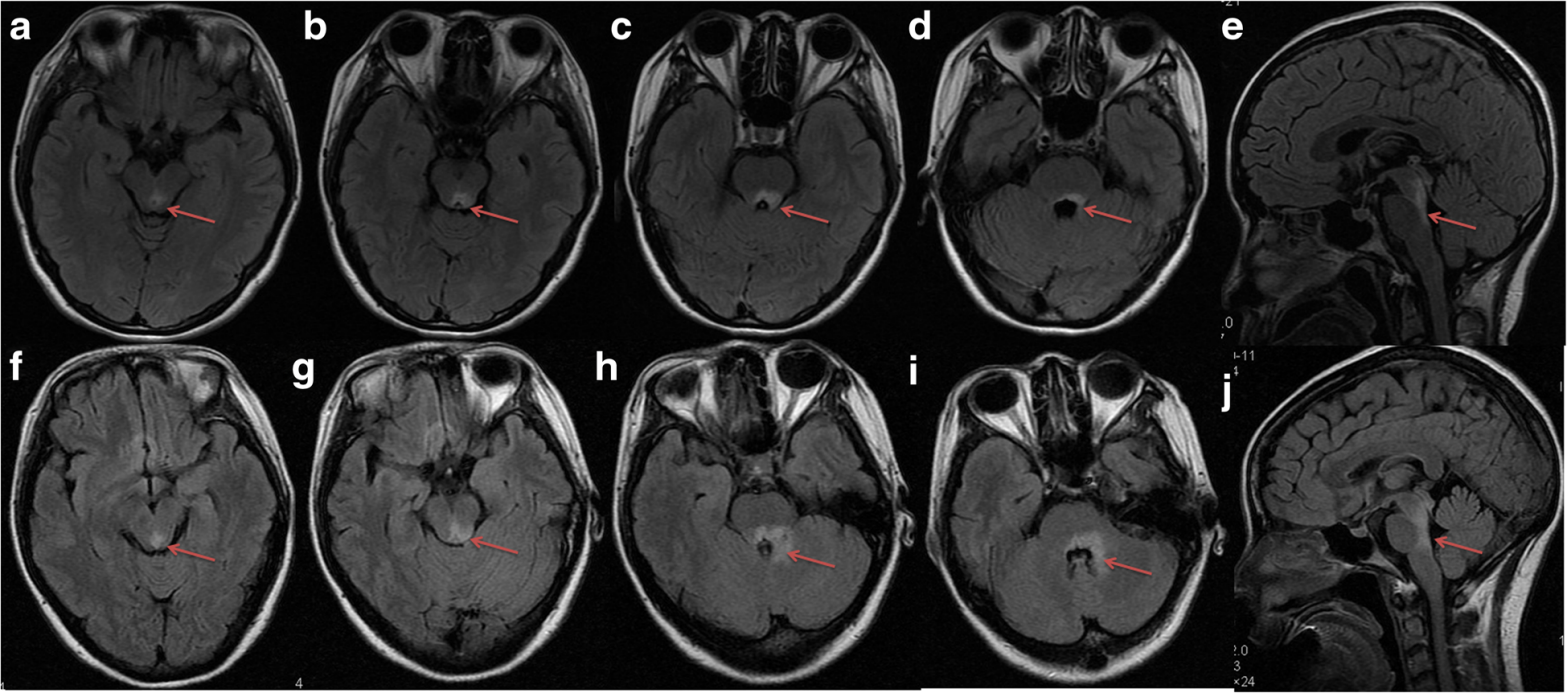
Axial T2-weighted FLAIR MRI shows periependymal lesions are involved in the dorsal midbrain (**a**, **b** and **f**, **g**; *arrow*) and pons (**c**, **d** and **h**, **i**; *arrows*). Sagittal T2-weighted FLAIR MRI shows increased signal surrounds the fourth ventricle (**e** and **j**; *arrows*)

Brain and spinal cord MRI examinations were conducted for all patients. On the sagittal section of brain MRI, the lesions were more frequently occurred in the medulla of anti-AQP4 (+) patients than those in anti-AQP4 (-) patients (78.75 % vs 35.29 %, *p* = 0.019). However, there were no statistical differences in the other parts of brainstem between these two groups, such as midbrain and pons (Fig. 2, Table 3). Dorsal-predominant lesions were involved more frequently in anti-AQP4 (+) patients than in the anti-AQP4 (-) patients (58.33 % vs 22.22 %, *p* = 0.01). The lesions in periependymal region were found more common in anti-AQP4 (+) patients than in anti-AQP4 (-) patients (35.29 % vs 7.14 %, *p* = 0.049) (Fig. 3, Table 3). And the lesions in area postrema were found in 4 anti-AQP4 (+) patients and 3 anti-AQP4 (-) patients (28.57 % vs 17.64 %, *p* = 0.383) (Fig. 2, Table 3). None of the spinal cord lesions were detected in the two groups when the initial episode of ABS. However, during the follow-up, segments of spinal cord lesions were longer in anti-AQP4 (+) patients than in anti-AQP4 (-) patients (5 vs 2, *p* = 0.001) (Table 4). LETM were also found more commonly in anti-AQP4 (+) patients (64.29 % vs 17.65 %, *p* = 0.012) at the duration of follow-up (Table 4).

**Table 3:** Comparison of brainstem MRI between anti-AQP4 (+) and anti-AQP4 (-) patients when the initial episode of ABS

MRIs	Anti-AQP4 (+)	Anti-AQP4 (-)	Total	*P* value
	*n* = 14	*n* = 17	*n* = 31	
Brainstem lesions				
Sagittal view				
Pons	6/14 (42.86 %)	8/17 (47.06 %)	14/31 (45.16 %)	0.551
Midbrain	3/14 (21.43 %)	5/17 (29.41 %)	8/31 (25.81 %)	0.466
Medulla	11/14 (78.57 %)	6/17 (35.29 %)	17/31 (54.84 %)	0.019
Axial view				
Dorsal	11/14 (78.57 %)	7/17 (41.17 %)	18/31 (58.06 %)	0.04
Ventral	3/14 (21.43 %)	10/17 (58.82 %)	13/31 (41.94 %)	0.04
Periependymal region	3/14 (21.43 %)	1/17 (5.88 %)	4/31 (12.90 %)	0.228
Area postrema	4/14 (28.57 %)	3/17 (17.64 %)	7/31 (22.58 %)	0.383

*ABS* acute brainstem syndrome

**Table 4:** Comparison of brain and spinalcord MRI between anti-AQP4 (+) and anti-AQP4 (-) patients during follow-up

MRIs	Anti-AQP4 (+)	Anti-AQP4 (-)	Total	*P* value
	*n* = 14	*n* = 17	*n* = 31	
Brain MRIs				
Lesions in other brain regions				
Juxtacortical	0/14 (0)	2/17 (11.76 %)	2/31 (6.45 %)	0.488
Subcortical	1/14 (7.14 %)	5/17 (29.41 %)	6/31 (19.35 %)	0.185
Infratentorial	14/14 (100 %)	17/17 (100 %)	31/31 (100 %)	
Spinal cord MRI				
Segments	5 (3–7)	2 (0–4)	3.5 (0–8)	0.001
Cervical	10/14 (71.43 %)	13/17 (76.47 %)	23/31 (74.19 %)	0.750
Thoracic	7/14 (50 %)	5/17 (29.41 %)	12/31 (38.71 %)	0.242
Cervical and thoracic	3/14 (21.43 %)	1/17 (5.88 %)	4/31 (12.90 %)	0.199
LETM	9/14 (64.29 %)	3/17 (17.65 %)	12/31 (38.71 %)	0.012
Meet Barkhof criteria	0/14 (0 %)	5/17 (29.41 %)	5/31 (16.13 %)	0.185

### CSF examinations

CSF specimens were obtained from all patients at the acute stage (Table 1). The protein concentrations in CSF of anti-AQP4 (+) patients (0.30 ± 0.10 mg/dl) were similar with those of anti-AQP4 (-) patients (0.25 ± 0.12 mg/dl). Anti-AQP4 (+) patients had a slightly higher number of white blood cells (WBCs) in CSF (11.29 ± 4.27) than anti-AQP4 (-) patients (8.47 ± 2.07). However, the values of IgG index in anti-AQP4 (+) patients were significantly higher than those of anti-AQP4 (-) patients (0.68 ± 0.07 vs 0.42 ± 0.13, *p* < 0.01). Only one anti-AQP4 (+) ABS patient and one anti-AQP4 (-) ABS patient were positive for OCBs in CSF.

### Prognosis in ABS patients

**Fig. 4:**
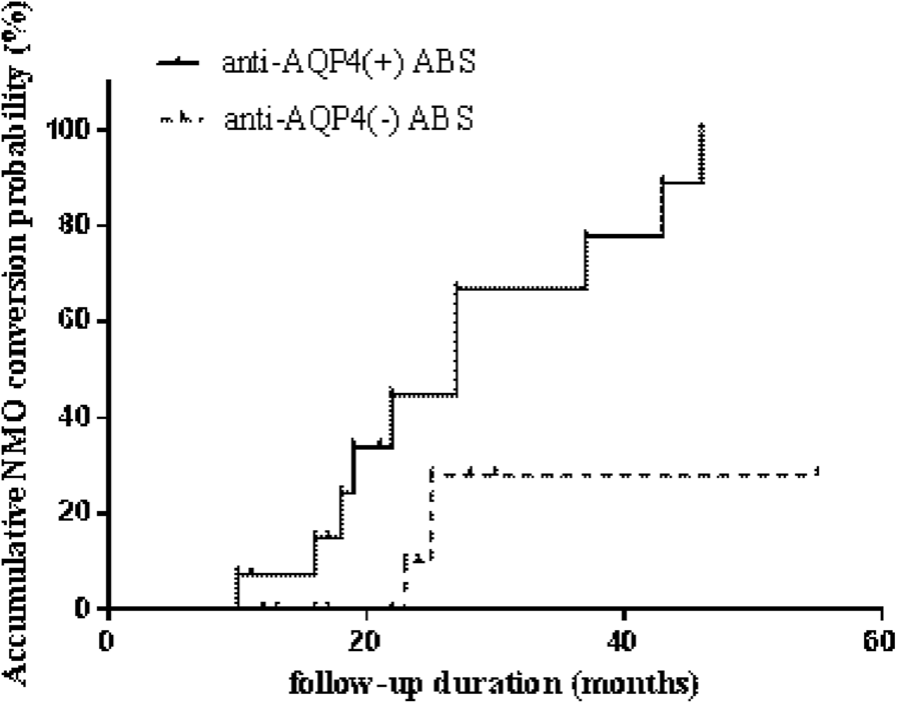
A survival analysis comparison of the risk of developing to NMO between anti-AQP4 (+) (*n* = 14) and anti-AQP4 (-) (*n* = 17) patients with ABS (*p* = 0.012)

All the patients were followed up at least 2 years after the first episode of ABS. The median duration of follow-up was 42 months (24–65 months). 7/31 (22.58 %) of patients did not show a second episode during the follow-up period, 12/31(38.70 %) of patients developed to NMO, 17/31(54.84 %) of patients converted to NMOSD, and 7/31(22.58 %) of patients converted to MS. In the follow-up duration, the mean annual relapse rates of anti-AQP4 (+) patients were significantly higher than those of anti-AQP4 (-) patients (1.05 ± 0.40 vs 0.72 ± 0.40, *p* = 0.031) (Table 1). Although the EDSS scores at the first attack were similar between two groups, anti-AQP4 (+) patients presented a significantly higher EDSS scores than anti-AQP4 (-) patients at the last visit of follow-up [5(3–7) VS 2.5(1.5–4.5), *p* < 0.01]. Kaplan-Meier survival analysis showed that the risk of developing to NMO in anti-AQP4 (+) patients were significantly higher than that in anti-AQP4 (-) patients (log rank 5.23, *p* = 0.012) (Fig. 4).

## Discussion

NMO frequently begins with an acute or subacute episode of ON or myelitis. However, brainstem symptoms in NMO were not rare [8, 10–12, 17]. According to the previous studies, brainstem symptoms have recently been described in NMO as well as in NMOSD [10, 12, 13, 17, 21]. In 17.05–30.61 % NMO patients, brainstem symptoms even act as the sole manifestation [14, 17]. “Brainstem symptoms with anti-AQP4 antibodies” has also been recommended in the latest diagnostic criteria for NMOSD [1]. Various brainstem symptoms such as area postrema clinical brainstem symptoms (including intractable hiccups, nausea and vomiting), diplopia, and bulbar dysfunctions attributed to lesions in the dorsal region of the medullar and the pons surrounding the fourth ventricle could be the first manifestation of symptoms [9, 10, 12, 14]. Our results demonstrated that area postrema clinical brainstem symptoms in anti-AQP4 (+) ABS patients were more frequent than those in anti-AQP4 (-) ABS patients. Intractable hiccups, nausea, and vomiting have been reported as unique symptoms in NMO due to the involvement of the pericanal region in the medulla oblongata, which included the areas postrema, nucleus tractus solitaries where the putative hiccup and vomiting centres are located [13, 14]. In our study, intractable hiccups, nausea, and vomiting occur in 71.43 % anti-AQP4 (+) ABS patients, which are more frequent than in anti-AQP4(-) ABS patients. Previous study also confirmed that intractable hiccups, nausea, and vomiting are clinical markers for the early phase of an exacerbation in NMO [22]. In addition, anti-AQP4 (+) ABS patients are prone to have recurrent brainstem symptoms before attacks of ON or myelitis.

In addition to clinical features, the distribution of brainstem lesions of anti-AQP4 (+) ABS patients also is different from anti-AQP4 (-) ABS patients. Although the midbrain and pons in the sagittal view are involved both in anti-AQP4 (+) ABS patients and anti-AQP4 (-) ABS patients, the medulla segments are more frequently involved in anti-AQP4 (+) ABS patients. In the axial view, anti-AQP4 (+) ABS patients present dorsal-predominant involvement, which is consistent with our previous report [23]. In AQP4 (+) ABS patients, majority of the lesions (78.57 %) are observed in the medulla including 28.57 % lesions occurring in the area postrema. There is an agreement between MRI findings and clinical presentation of brainstem lesions. This study supports that the brainstem, especially medulla and area postrema, is the important region of attack in NMO [11, 17].

NMO-IgG has been proved to be highly specific and moderately sensitive to NMO. The prevalence of anti-AQP4 antibodies in NMO patients ranged from 50–60 % [3, 24–26]. However, NMO-IgG is not restricted to these patients fulfilling all criteria for a definite diagnosis of NMO [27–29]. This antibody is also identified in partial syndromes, the frequency of anti-AQP4 in acute partial transverse myelitis is 4.5 % [30], while 26.9 % in recurrent ATM [31], increasing to 37.9–60 % in LETM [27, 31, 32] and 20 % in recurrent ON [33], 5.8 % in acute monosymptomatic ON [34]. Our study provided that the frequency of anti-AQP4 in ABS is 45.16 %. Currently, sensitive marker(s) to predict the conversion of ABS to NMO are still absent. Our results found that 100 % of the anti-AQP4 (+) ABS patients experience clinical relapse, and 71.43 % of anti-AQP4 (+) ABS patients convert to NMO in the following 3 years, while only 11.76 % of the anti-AQP4 (-) ABS patients convert to NMO, and the cumulative NMO conversion probability is significantly higher in anti-AQP4 (+) ABS patients than that in anti-AQP4 (-) ABS patients. These results are similar to those patients with ON or LETM as the initial presentation, which also demonstrated a rapid NMO conversion in the first 2 years after the initial episode [32, 35]. The results suggested that anti-AQP4 antibodies may be useful for diagnosis and prognosis of patients who presented with isolated ABS. Moreover, 29.42 % of anti-AQP4 (+) ABS patients experience recurrent brainstem symptoms before attack of ON or myelitis, which suggested that anti-AQP4 antibody associated recurrent brainstem symptoms should be paid more attention. The anti-AQP4 (+) ABS patients are at high risk for development of severe disability.

Several studies have also identified a new antigenic target, myelin oligodendrocytic glycoprotein (MOG), as being of interest in seronegative AQP4 patients [36]. Compared with AQP4-positivity patients, serum MOG antibodies do have a distinct clinical phenotype from AQP4-positivity NMO that is characterized by fewer relapses, a better clinical outcome, and a wider spectrum of MRI features, which may suggest a sort of disease distinct from NMO or MS [37–39]. This antibody is found in around 20 % of AQP4-seronegative patients. MOG testing was not performed in this study, therefore we can’t exclude the bias of MOG-seropositive patients in our AQP4 negative cohort. However, brainstem lesions, which are a hallmark of AQP4-seropositive NMO/NMOSD, occur less frequently in MOG-seropositive patients [38, 40]. Moreover, the primary endpoint of our study is the conversion of typical NMO. Therefore, this would make little impact on our results.

Therapy of NMO should be initiated early. Azathioprine and rituximab are suggested as first-line treatments [41]. In patients with NMOSD, IFN-β treatment [42], glatiramer acetate [43], natalizumab [44] and fingolimod (FTY720) [45] are ineffective for preventing relapses or fail to control disease activity. In our study, no patients received the above treatments, which might have treatment effects on accumulative NMO conversion probability. Therefore, the risk of conversion to NMO in anti-AQP4 (+) ABS patients seems higher in the first 2 years based on this study. We recommend that all patients with ABS make test for NMO-IgG and that seropositive patients receive immunosuppressive treatments which are effective in reducing NMO relapse frequency, such as azathioprine or rituximab.

## Conclusions

ABS represents an inaugural or limited form of NMO in a high proportion of anti-AQP4 (+) patients.

## Abbreviations

ABS: Acute brainstem syndrome
AQP4: Aquaporin 4
CSF: Cerebrospinal fluid
EDSS: Expanded Disability Status Scale
MRI: Magnetic resonance imaging
NMO: Neuromyelitis optica
NMOSD: NMO spectrum disorder
OCB: Oligoclonal bands

## References

[1] Wingerchuk DM, Banwell B, Bennett JL, Cabre P, Carroll W, Chitnis T, de Seze J, Fujihara K, Greenberg B, Jacob A, Jarius S, Lana-Peixoto M, Levy M, Simon JH, Tenembaum S, Traboulsee AL, Waters P, Wellik KE, Weinshenker BG (2015). International Panel for NMO Diagnosis. International consensus diagnostic criteria for neuromyelitis optica spectrum disorders. Neurology.

[2] Wingerchuk DM, Hogancamp WF, O’Brien PC, Weinshenker BG (1999). The clinical course of neuromyelitis optica (Devic’s syndrome). Neurology.

[3] Lennon VA, Wingerchuk DM, Kryzer TJ, Pittock SJ, Lucchinetti CF, Fujihara K, Nakashima I, Weinshenker BG (2004). A serum autoantibody marker of neuromyelitis optica: distinction from multiple sclerosis. Lancet.

[4] McFarling DA, Susac JO (1979). Hoquet diabolique: intractable hiccups as a manifestation of multiple sclerosis. Neurology.

[5] Pittock SJ, Lennon VA, Krecke K, Wingerchuk DM, Lucchinetti CF, Weinshenker BG (2006). Brain abnormalities in neuromyelitis optica. Arch Neurol.

[6] Misu T, Fujihara K, Nakashima I, Miyazawa I, Okita N, Takase S, Itoyama Y (2002). Pure optic-spinal form of multiple sclerosis in Japan. Brain.

[7] Kim HJ, Paul F, Lana-Peixoto MA, Tenembaum S, Asgari N, Palace J, Klawiter EC, Sato DK, de Seze J, Wuerfel J, Banwell BL, Villoslada P, Saiz A, Fujihara K, Kim SH (2015). Guthy-Jackson Charitable Foundation NMO International Clinical Consortium & Biorepository. MRI characteristics of neuromyelitis optica spectrum disorder: an international update. Neurol.

[8] Wang Y, Zhang L, Zhang B, Dai Y, Kang Z, Lu C, Qiu W, Hu X, Lu Z (2014). Comparative clinical characteristics of neuromyelitis optica spectrum disorders with and without medulla oblongata lesions. J Neurol.

[9] Li Y, Jiang B, Chen B, Zhao M, Zhou C, Wang S, Li J, Wang R. Neuromyelitis optica spectrum disorders with multiple brainstem manifestations: a case report. Neurol Sci. 2015; Apr 3. [Epub ahead of print] 10.1007/s10072-015-2196-z 25837714

[10] Kremer L, Mealy M, Jacob A, Nakashima I, Cabre P, Bigi S, Paul F, Jarius S, Aktas O, Elsone L, Mutch K, Levy M, Takai Y, Collongues N, Banwell B, Fujihara K, de Seze J (2014). Brainstem manifestations in neuromyelitis optica: a multicenter study of 258 patients. Mult Scler.

[11] Chan KH, Tse CT, Chung CP, Lee RL, Kwan JS, Ho PW, Ho JW (2011). Brain involvement in neuromyelitis optica spectrum disorders. Arch Neurol.

[12] Wang KC, Lee CL, Chen SY, Lin KH, Tsai CP (2011). Prominent brainstem symptoms/signs in patients with neuromyelitis optica in a Taiwanese population. J Clin Neurosci.

[13] Misu T, Fujihara K, Nakashima I, Sato S, Itoyama Y (2005). Intractable hiccup and nausea with periaqueductal lesions in neuromyelitis optica. Neurology.

[14] Popescu BF, Lennon VA, Parisi JE, Howe CL, Weigand SD, Cabrera-Gómez JA, Newell K, Mandler RN, Pittock SJ, Weinshenker BG, Lucchinetti CF (2011). Neuromyelitis optica unique area postrema lesions: nausea, vomiting, and pathogenic implications. Neurology.

[15] Jarius S, Ruprecht K, Wildemann B, Kuempfel T, Ringelstein M, Geis C, Kleiter I, Kleinschnitz C, Berthele A, Brettschneider J, Hellwig K, Hemmer B, Linker RA, Lauda F, Mayer CA, Tumani H, Melms A, Trebst C, Stangel M, Marziniak M, Hoffmann F, Schippling S, Faiss JH, Neuhaus O, Ettrich B, Zentner C, Guthke K, Hofstadt-van Oy U, Reuss R, Pellkofer H, Ziemann U, Kern P, Wandinger KP, Bergh FT, Boettcher T, Langel S, Liebetrau M, Rommer PS, Niehaus S, Münch C, Winkelmann A, Zettl UUK, Metz I, Veauthier C, Sieb JP, Wilke C, Hartung HP, Aktas O, Paul F (2012). Contrasting disease patterns in seropositive and seronegative neuromyelitis optica: A multicentre of 175 patients. J Neuroinflammation.

[16] Niino M, Uesugi H, Takahashi T, Fukazawa T, Minami N, Tashiro J, Fujiki N, Doi S, Kikuchi S (2012). Recurrent brainstem lesions mimicking infarctions in an elderly patient with neuromyelitis optica spectrum disorder. Intern Med.

[17] Asgari N, Skejoe HP, Lillevang ST, Steenstrup T, Stenager E, Kyvik KO (2013). Modifications of longitudinally extensive transverse myelitis and brainstem lesions in the course of neuromyelitis optica (NMO): a population-based, descriptive study. BMC Neurol.

[18] Wingerchuk DM, Lennon VA, Pittock SJ, Lucchinetti CF, Weinshenker BG (2006). Revised diagnostic criteria for neuromyelitis optica. Neurology.

[19] Polman CH, Reingold SC, Banwell B, Clanet M, Cohen JA, Filippi M, Fujihara K, Havrdova E, Hutchinson M, Kappos L, Lublin FD, Montalban X, O’Connor P, Sandberg-Wollheim M, Thompson AJ, Waubant E, Weinshenker B, Wolinsky JS (2011). Diagnostic criteria for multiple sclerosis: 2010 revisions to the McDonald criteria. Ann Neurol.

[20] Kurtzke JF (1983). Rating neurologic impairment in multiple sclerosis: an expanded disability status scale (EDSS). Neurology.

[21] Chalumeau-Lemoine L, Chretien F, Gaëlle Si Larbi A, Brugieres P, Gray F, Brun-Buisson C, Creange A (2006). Devic disease with brainstem lesions. Arch Neurol.

[22] Takahashi T, Miyazawa I, Misu T, Takano R, Nakashima I, Fujihara K, Tobita M, Itoyama Y (2008). Intractable hiccup and nausea in neuromyelitis optica with anti-aquaporin-4 antibody: a herald of acute exacerbations. J Neurol Neurosurg Psychiatry.

[23] Lu Z, Zhang B, Qiu W, Kang Z, Shen L, Long Y, Huang J, Hu X (2011). Comparative brain stem lesions on MRI of acute disseminated encephalomyelitis, neuromyelitis optica, and multiple sclerosis. PLoS One.

[24] Marignier R, De Sèze J, Vukusic S, Durand-Dubief F, Zéphir H, Vermersch P, Cabre P, Cavillon G, Honnorat J, Confavreux C (2008). NMO-IgG and Devic’s neuromyelitis optica: a French experience. Mult Scler.

[25] Saiz A, Zuliani L, Blanco Y, Tavolato B, Giometto B, Graus F (2007). Revised diagnostic criteria for neuromyelitis optica (NMO). Application in a series of suspected patients. J Neurol.

[26] Paul F, Jarius S, Aktas O, Bluthner M, Bauer O, Appelhans H, Franciotta D, Bergamaschi R, Littleton E, Palace J, Seelig HP, Hohlfeld R, Vincent A, Zipp F (2007). Antibody to aquaporin 4 in the diagnosis of neuromyelitis optica. PLoS Med.

[27] Weinshenker BG, Wingerchuk DM, Vukusic S, Linbo L, Pittock SJ, Lucchinetti CF, Lennon VA (2006). Neuromyelitis optica IgG predicts relapse after longitudinally extensive transverse myelitis. Ann Neurol.

[28] Jarius S, Franciotta D, Bergamaschi R, Wright H, Littleton E, Palace J, Hohlfeld R, Vincent A (2007). NMO-IgG in the diagnosis of neuromyelitis optica. Neurology.

[29] Matsuoka T, Matsushita T, Kawano Y, Osoegawa M, Ochi H, Ishizu T, Minohara M, Kikuchi H, Mihara F, Ohyagi Y, Kira J (2007). Heterogeneity of aquaporin-4 autoimmunity and spinal cord lesions in multiple sclerosis in Japanese. Brain.

[30] Scott TF, Kassab SL, Pittock SJ (2006). Neuromyelitis optica IgG status in acute partial transverse myelitis. Arch Neurol.

[31] Alvarenga MP, Alvarenga RM, Alvarenga MP, Santos AM, Thuler LC (2012). Anti-AQP(4) antibody in idiopathic acute transverse myelitis with recurrent clinical course: frequency of positivity and influence in prognosis. J Spinal Cord Med.

[32] Chang KH, Lyu RK, Chen CM, Wu YR, Chang HS, Huang CC, Kuo HC, Chu CC, Hsu WC, Ro LS (2013). Distinct features between longitudinally extensive transverse myelitis presenting with and without anti-aquaporin 4 antibodies. Mult Scler.

[33] Matiello M, Lennon VA, Jacob A, Pittock SJ, Lucchinetti CF, Wingerchuk DM, Weinshenker BG (2008). NMO-IgG predicts the outcome of recurrent optic neuritis. Neurology.

[34] Jarius S, Frederikson J, Waters P, Paul F, Akman-Demir G, Marignier R, Franciotta D, Ruprecht K, Kuenz B, Rommer P, Kristoferitsch W, Wildemann B, Vincent A (2010). Frequency and prognostic impact of antibodies to aquaporin-4 in patients with optic neuritis. J Neurol Sci.

[35] Cabrera-Gómez JA, Bonnan M, González-Quevedo A, Saiz-Hinarejos A, Marignier R, Olindo S, Graus F, Smadja D, Merle H, Thomas L, Gómez-García A, Cabre P (2009). Neuromyelitis optica positive antibodies confer a worse course in relapsing-neuromyelitis optica in Cuba and French West Indies. Mult Scler.

[36] Kim SM, Woodhall MR, Kim JS (2015). Antibodies to MOG in adults with inflammatory demyelinating disease of the CNS. Neurol Neuroimmunol Neuroinflamm.

[37] Kitley J, Waters P, Woodhall M, Leite MI, Murchison A, George J, Küker W, Chandratre S, Vincent A, Palace J (2014). Neuromyelitis optica spectrum disorders with aquaporin-4 and myelin-oligodendrocyte glycoprotein antibodies: a comparative study. JAMA Neurol.

[38] Sato DK, Callegaro D, Lana-Peixoto MA, Waters PJ, de Haidar Jorge FM, Takahashi T, Nakashima I, Apostolos-Pereira SL, Talim N, Simm RF, Lino AM, Misu T, Leite MI, Aoki M, Fujihara K (2014). Distinction between MOG antibodypositive and AQP4 antibody-positive NMO spectrum disorders. Neurology.

[39] Probstel AK, Rudolf G, Dornmair K, Collongues N, Chanson JB, Sanderson NS, Lindberg RL, Kappos L, de Seze J, Derfuss T (2015). Anti-MOG antibodies are present in a subgroup of patients with a neuromyelitis optica phenotype. J Neuroinflammation.

[40] Dale RC, Tantsis EM, Merheb V, Kumaran RY, Sinmaz N, Pathmanandavel K, Ramanathan S, Booth DR, Wienholt LA, Prelog K, Clark DR, Guillemin GJ, Lim CK, Mathey EK, Brilot F (2014). Antibodies to MOG have a demyelination phenotype and affect oligodendrocyte cytoskeleton. Neurol Neuroimmunol Neuroinflamm.

[41] Trebst C, Jarius S, Berthele A, Paul F, Schippling S, Wildemann B, Borisow N, Kleiter I, Aktas O, Kümpfel T, Neuromyelitis Optica Study Group (NEMOS) (2014). Update on the diagnosis and treatment of neuromyelitis optica: recommendations of the Neuromyelitis Optica Study Group (NEMOS). J Neurol.

[42] Kim SH, Kim W, Li XF, Jung IJ, Kim HJ (2012). Does interferon beta treatment exacerbate neuromyelitis optica spectrum disorder?. Mult Scler.

[43] Ayzenberg I, Schöllhammer J, Hoepner R, Hellwig K, Ringelstein M, Aktas O, Kümpfel T, Krumbholz M, Trebst C, Paul F, Pache F, Obermann M, Zeltner L, Schwab M, Berthele A, Jarius S, Kleiter I, Neuromyelitis Optica Study Group (NEMOS) (2016). Efficacy of glatiramer acetate in neuromyelitis optica spectrum disorder: a multicenter retrospective study. J Neurol.

[44] Kleiter I, Hellwig K, Berthele A, Kümpfel T, Linker RA, Hartung HP, Paul F, Aktas O, Neuromyelitis Optica Study Group (2012). Failure of natalizumab to prevent relapses in neuromyelitis optica. Arch Neurol.

[45] Min JH, Kim BJ, Lee KH (2012). Development of extensive brain lesions following fingolimod (FTY720) treatment in a patient with neuromyelitis optica spectrum disorder. Mult Scler.

